# The Sexual Dimorphic Synapse: From Spine Density to Molecular Composition

**DOI:** 10.3389/fnmol.2022.818390

**Published:** 2022-02-18

**Authors:** Mara Uhl, Michael J. Schmeisser, Sven Schumann

**Affiliations:** ^1^Institute for Microscopic Anatomy and Neurobiology, University Medical Center of the Johannes Gutenberg University Mainz, Mainz, Germany; ^2^Focus Program Translational Neurosciences, University Medical Center of the Johannes Gutenberg University Mainz, Mainz, Germany

**Keywords:** sexual dimorphism, synapse, spine density, estrogen, estrogen receptor, mosaic brain

## Abstract

A synaptic sexual dimorphism is relevant in the context of multiple neurodevelopmental, neurodegenerative, and neuropsychiatric disorders. Many of these disorders show a different prevalence and progression in woman and man. A similar variance is also present in corresponding animal models. To understand and characterize this dimorphism in pathologies it is important to first understand sex differences in unaffected individuals. Therefore, sexual differences have been studied since 1788, first focusing on brain weight, size, and volume. But as these measures are not directly related to brain function, the investigation of sexual dimorphism also expanded to other organizational levels of the brain. This review is focused on sexual dimorphism at the synaptic level, as these specialized structures are the smallest functional units of the brain, determining cell communication, connectivity, and plasticity. Multiple differences between males and females can be found on the levels of spine density, synaptic morphology, and molecular synapse composition. These differences support the importance of sex-disaggregated data. The specificity of changes to a particular brain region or circuit might support the idea of a mosaic brain, in which each tile individually lies on a continuum from masculinization to feminization. Moreover, synapses can be seen as the smallest tiles of the mosaic determining the classification of larger areas.

## Introduction

Sexual dimorphism is the difference in appearance between males and females of the same species. Many neurodevelopmental, neurodegenerative and neuropsychiatric disorders, among them, autism spectrum disorder (ASD) (Lai et al., 2013; Werling and Geschwind, 2013; Baio et al., 2018), stress disorders (Ramikie and Ressler, 2018), anxiety disorders (Maeng and Milad, 2015; Asher et al., 2017), depression (Kessler et al., 1993; Altemus, 2006; Eid et al., 2019), schizophrenia (Jackson et al., 2013; Mendrek and Mancini-Marïe, 2016), Alzheimer’s disease (Irvine et al., 2012; Association, 2016) and Parkinson’s disease [reviewed in Gillies et al. (2014), Hirsch et al. (2016)] show different prevalence and/or clinical characteristics in woman and man. Similar sexual differences are also imminent in corresponding model organisms. Even if the reasons for these differences are not fully understood yet, they can at least partially be attributed to a sexual dimorphic brain. To understand the role of the dimorphic brain in disorders, it is important to first investigate sex differences in unaffected individuals. For this, different approaches and techniques have been used over the past decades.

Early on studying the brain aimed to explain, so thought, given behavioral and intellectual differences between woman and man (Schröter et al., 2012). Soemmerring (1788) reported in his neuroanatomical studies that the male brain is larger and heavier than the female brain. This statement was later on specified by the anatomist Tiedemann, who showed that, in correlation to body weight and size, the female brain is larger and of higher weight (Tiedemann, 1837). Since these early publications a lot of work has been done in the field of the brain sexual dimorphism. Regarding brain size, a more in-depth analysis not only examined the overall brain size, but the volume of different brain regions, gray and white matter. This showed the existence of region and sex-specific volumetric differences (Gur et al., 1999; Ruigrok et al., 2014). Even considering these advanced methods, it still proves to be difficult to relate brain size to behavioral differences. Therefore, it is important to investigate the sexual dimorphism of the brain on different organizational levels.

The lowest functional and organizational level of the brain are synapses. They facilitate the communication between neurons and thereby determine functions and mechanisms, from learning and memory formation to many pathomechanisms (van Spronsen and Hoogenraad, 2010; Mayford et al., 2012; Verpelli et al., 2012; Lisman et al., 2018; Batool et al., 2019). Considering this important role of synapses, relatively little is known about synaptic sex differences. Till now research in this field often focuses on one sex, mainly males, leaving the transferability of the results undetermined. Consequently, we see the importance to review the data on the synaptic sexual dimorphism and evaluate the importance of sex-disaggregated data in this context. To provide a comprehensive overview of sex-specific synaptic dimorphism, this review includes information on spine and synapse density, morphology, and molecular composition.

## Spine and Synapse Density

Options to study the abundance of synapses in the context of sex differences are to either look at the density of synapses directly, or to investigate spine density as a proximate of synapse density. Diverse attempts using both methods have been undertaken in animal models. Early studies were performed in Sprague-Dawley rats. These studies did not focus on intersex differences, they reported changes of the spine density in pyramidal cells of the hippocampus during the female estrus cycle and the effect of gonadal steroids in adult [postnatal day 110 (P110)] rats. This showed that the spine density is highest in proestrus and lowest in estrus (Woolley et al., 1990). Ovarectomization and rescue experiments revealed the dependency of this effect on the gonadal hormones estrogen and progesterone (P75) (Gould et al., 1990). Comparing 2–3 months old female Sprague-Dawley rats in proestrus to male rats, the female rats have a higher density of apical dendritic spines of hippocampal CA1 pyramidal cells. These cyclical as well as intersex differences lead to different reactions to stress. While in female rats in proestrus the spine density decreases, it increases in males and non-proestrus females. The spines were analyzed using Golgi impregnation (Shors et al., 2001). Putative orexin neurons exhibit a higher spine density in female mice than in male mice at P65 to P75 (Grafe et al., 2019). At basal dendrites of the CA1 region, the spine count is higher in female Sprague-Dawley rats at 7 weeks after birth (Bowman et al., 2014). No variation in spine count was found in CA1 apical dendritic spines, neither within the estrus cycle nor between the sexes. In contrast to that, the spine size deviates. Female mice have more large spines (head diameter ≥ 0.6 μm) compared to males at 8 weeks. This difference is also imminent in primary hippocampal neurons created from male and female Wistar rats after birth (P0), but it is not visible in cultures generated before birth (E18). This indicates a sex-specific maturation of the neurons between these time points (Brandt et al., 2020). The age dependency of sex differences is also visible in the stratum radiatum (SR) of the hippocampal CA1 region. In this region female C57BL/6J mice have a higher density of dendritic spines and synaptic boutons at P15 than male mice, also resulting in a higher overall synapse count in females. This difference diminishes with aging and is not imminent at P40 (Weinhard et al., 2018). Overall, most, but not all studies in the hippocampus report a higher spine density in females compared to males.

**FIGURE 1:**
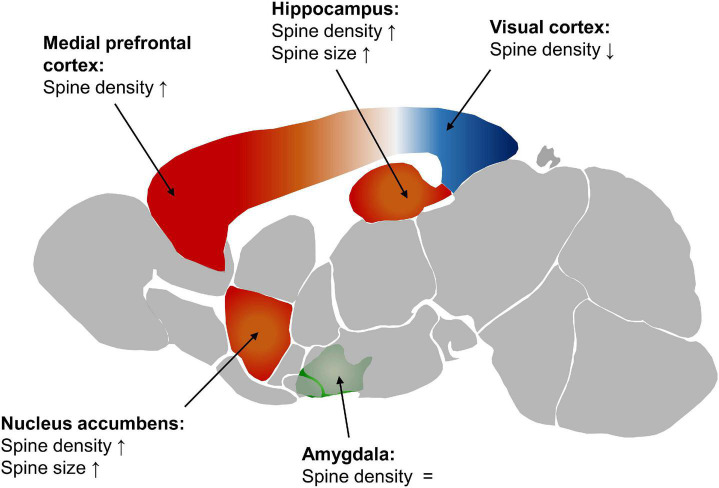
Spine density and size in the female mouse brain compared to the male. Red displays a higher spine density in females, blue a lower one, and green similar levels in males and females. The depiction of the mouse brain is modified from the Allen Mouse Brain Atlas (2004), Lein et al. (2007).

Differences in spine density also occur in other brain regions (Figure 1) and can be persistent over the course of aging. In pyramidal cells of the auditory and visual regions of the cortex (A1, A2, V1, V2, and TeA) the dendritic spine density is higher in male C57BL/6 J mice than in females. This is persistent from P28 to P84 (Parker et al., 2020). Looking in detail at layer 5 of the primary visual cortex, the difference in spine density disappears by P60 (Muñoz-Cueto et al., 1991).

In layer II/III of the prelimbic portion of the medial prefrontal cortex (PFC) 7 weeks old male Sprague-Dawley rats have fewer spines on the basal tree of the neurons than females (Bowman et al., 2014).

In the nucleus accumbens first analysis showed a higher spine density and larger spines in female Sprague-Dawley rats at P60 to P67. Later on, this was specified by a study showing a rostro-caudal gradient in female mice with a higher spine density in the caudal core of the nucleus accumbens and a higher tendency toward larger spines in the rostral core (Forlano and Woolley, 2010; Wissman et al., 2012).

Investigating adult (3 months) Wistar rats no differences in dendritic spine density were found in the posterodorsal medial amygdaloid nucleus (MePD) between diestrus females and males (Arpini et al., 2010). Another study on 3 months old male Wistar rats showed that the number of inhibitory synapses on dendritic shafts of right MePD varies with the estrus cycle, being higher in proestrus than in any other phase and in male mice. Regarding synaptic vesicles in the MePD it was shown that on the right side, females have higher numbers of vesicles throughout all stages of the estrus cycle than males, which do not show a left-right difference. Furthermore, in proestrus, females have more docked vesicles on the right side (Brusco et al., 2014). In the basolateral nucleus (BL) of the amygdala the number of spine synapses is higher in female C57BL/6J than in males at P65 (Bender et al., 2017). In juvenile, 25–29 days old, Sprague-Dawley rats, males have a greater frequency of miniature excitatory postsynaptic currents (EPSC) in the left hemisphere of the MePD than females, while no difference occurs in miniature EPSC amplitude, indicating a higher number of excitatory synapses. This was further supported by a higher number of asymmetric synapses per neuron in males. These asymmetric synapses were classified as excitatory synapses due to a lack of GABA immunostaining. In this experiment the number of symmetric synapses was not changed between the sexes (Cooke and Woolley, 2005).

Spine and synapse density are difficult to access in humans due to the need of fresh brain tissue of corresponding brain regions from several individuals. Therefore, corresponding measurements and specifically comparative studies are rare. The sample numbers tend to be low in such experiments, using biopsy or autopsy material. In addition, samples are often limited to pathological conditions (e.g., epilepsy). Moreover, some of the existing studies contradict each other. A higher synapse density has been shown in man compared to woman in all layers of the temporal neocortex by Alonso-Nanclares et al. (2008). Contradicting to that, another study reported the synapse density in layer 4 of the temporal lobe neocortex to be about 2- to 3-folds higher in females than in males. In this study the overall inter-individual variability of synapse/mm^3^ was reported to be between 1.5 and 4-folds, which raises the question if the reported male to female difference is conclusive (Yakoubi et al., 2019). New methods for in vivo imaging could shine light on the investigation of spine/synapse density in humans in the future (Finnema et al., 2016).

Spine and synapse density are dependent on underlying molecular mechanisms determining spine formation and synapse plasticity. The fact that spine and synapse density deviate between the sexes implicates a dimorphism in synaptic composition and signaling pathways involved in these processes. Consequently, the following parts of this review focus on known molecular sex differences of synapses.

## Molecular Synapse Composition

There are different ways of investigating the molecular composition of synapses. The analysis of protein or RNA abundance allows a broad overview, which can give starting points for further in-depth analysis. Focusing on specific proteins or pathways allows a deeper understanding of certain processes. For the investigation of sex differences both approaches are important and complement each other.

In an RNA-seq analysis of the hippocampus from female and male mice of different ages (1, 2, and 4 months), 198 transcripts differed between sexes in at least one age group. Sixty-eight mRNAs were found, which change their expression in a sex-dependent manner over time. The enriched transcripts are involved in protein folding and response to unfolded protein as identified by a GO analysis (Bundy et al., 2017). Unfortunately, this analysis was not synapse specific and therefore only shows broad differences. In the rat medial amygdala (MeA), levels of different microRNAs that affect synaptic plasticity, vary with the estrus cycle and are differentially expressed in males and females (Hirsch et al., 2018). In another RNA-seq analysis no differences were found in major cell types, relative abundance and major cell type markers. Looking more in detail at neurons, more sex-specific differentially expressed genes were found in GABAergic neurons than in glutamatergic neurons. Because of that a linear discriminative analysis (STAR Methods) is able to classify male vs. female samples based on the expression profile of neuronally enriched genes if the analysis program is trained on GABAergic cells, but not if it is trained on glutamatergic neurons (Chen et al., 2019). A more synapse-specific analysis was performed with synapse-enriched fractions of different brain regions. This comparative proteome analysis among different brain regions in male and female C57BL/6J mice at P42 showed differential expression of proteins between sexes in all of them, without controlling for the estrus stage. Seventy-one proteins were found to be changed between males and females in the hippocampus, 28 in the cerebellum, 7 in the striatum, and 8 in the cortex. Among them were proteins related to ASD: DDX3X, KMT2C, MYH10, and SET (Distler et al., 2020). DDX3X is primarily associated with intellectual disability in woman (Snijders Blok et al., 2015; Wang X. et al., 2018). It regulates embryonic corticogenesis by affecting neurogenesis in the cortex. A missense mutation in this gene can lead to an altered translation of selected mRNAs and to the accumulation of ribonucleoprotein granules (Lennox et al., 2020). KMT2C is a histone H3 lysine 4 methyltransferase (Froimchuk et al., 2017) and involved in neuronal differentiation (Dhar et al., 2012). MYH10 encodes for the non-muscle myosin heavy chain IIB, which is involved in brain development, influencing the migration of specific neurons (Ma et al., 2004). SET has also been related to intellectual disability (Stevens et al., 2018). It is a nuclear proto-oncogene (von Lindern et al., 1992) and encodes for a component of a histone acetyltransferase inhibitor (Seo et al., 2001). Although most of these proteins have known neuronal implications, their synaptic functions are unknown and remain to be further investigated. This indicates the relevance of differentially expressed proteins especially in ASD, but also in the overall context of neuropsychiatric disorders (Distler et al., 2020).

Aside from these RNA and proteome-focused approaches, also single proteins and pathways have been investigated focusing on a sexual dimorphism. These studies sometimes show differences, which are not detected with broader techniques. Therefore, the next part will focus on these case studies of specific proteins and pathways.

### Estradiol Pathway

Most differences in synaptic signaling currently known are related to sex steroids, which are produced in the gonads, the adrenal glands, the placenta as well as directly in the brain (Kimoto et al., 2001; Kawato et al., 2002; Prange-Kiel et al., 2003; Hojo et al., 2004; Woolley, 2007).

One of these gonadal steroids is estradiol. It is accessible systemically, but fast concentration changes, which have been observed in the brain, do not match up with the systemic fluctuations. This can be explained with estradiol being produced in different brain regions of both female and male mice. The regions include the hippocampus, cortical regions, the bed nucleus of the stria terminalis, the MePD, the medial preoptic area (MPOA) and discrete hypothalamic regions (Lauber and Lichtensteiger, 1994; Wagner and Morrell, 1996; Woolley, 2007; Cornil, 2018). The synthesis of estrogens from androgens is catalyzed by aromatase (Boon et al., 2010).

Estradiol was shown to influence a variety of processes and pathways (Morley et al., 1992; Aronica et al., 1994; Webb et al., 1995; Gu and Moss, 1996; Mermelstein et al., 1996; Watters and Dorsa, 1998; Singer et al., 1999; Wade et al., 2001; Boulware et al., 2005; Kramár et al., 2009). It is known to influence synaptic plasticity and increases EPSC amplitude in both sexes (Smejkalova and Woolley, 2010; Jain et al., 2019). Inhibition of cytochrome P450 aromatase (AROM), which converts testosterone to 17β-estradiol (E2), diminishes long-term potentiation (LTP) in female rat acute slices of the BL of the amygdala, while not significantly affecting LTP in male acute slices. Moreover, the inhibition reduces the density of spine synapses in the BL, by reducing the spine number in females (Bender et al., 2017). A similar effect is observed in CA1 SR hippocampal neurons, which lose spines and spine synapses after aromatase inhibition in females (Kretz et al., 2004; Zhou et al., 2010; Vierk et al., 2012). In the PFC, the level of AROM is higher in females than in males. Moreover, inhibition of AROM diminishes the protective effect estrogen has in females against repeated stress (Wei et al., 2014). Effects of AROM have also been reported in humans. Women treated with AROM inhibitors reported memory deficits, which could be connected to effects on the hippocampus (Shilling et al., 2003; Bayer et al., 2015), and symptoms of depression, possibly related to changes in the amygdala (Gallicchio et al., 2012). One of the mechanisms through which E2 influences LTP in females is depending on Protein Kinase A (PKA). The inhibition of PKA diminishes the potentiation of EPSCs by E2 in CA1 pyramidal cells of hippocampal slice cultures from female Sprague-Dawley rats. Thereby, blocking of PKA also inhibits LTP in females. In males, a combined inhibition of PKA and Ca^2+^/calmodulin-dependent protein kinase II (CaMKII) is necessary to block EPSC potentiation. Moreover, CaMKII is necessary for the maintenance of potentiation in both sexes. Other kinases – mitogen-activated protein kinase, Src tyrosine kinase, and rho-associated kinase – influence initial EPSC potentiation in both sexes, but are not involved in maintenance. Further investigating the role of calcium, it was shown that blocking of L-type calcium channels in combination with depletion of internal calcium stores diminishes potentiation of EPSCs in male Sprague-Dawley rat CA1 pyramidal cells. In females one of both factors is sufficient to lead to the same result (Jain et al., 2019).

**FIGURE 2:**
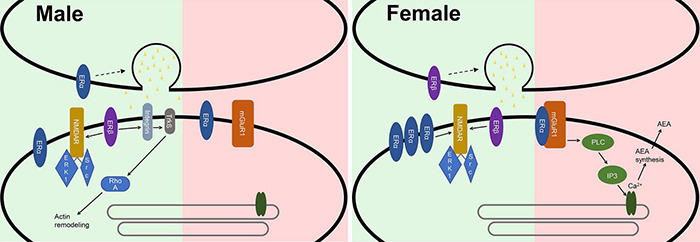
Synaptic differences of male and female synapses in the hippocampus. In excitatory synapses (green) ERα is more abundant in females, where it is involved in the LTP enhancing effects of E2. In males ERα could be involved in presynaptic mechanisms of glutamate release. ERβ sparks a presynaptic response leading to the release of glutamate in females, while it postsynaptically modulates the actin cytoskeleton in males. In inhibitory synapses (red) E2 promotes an interaction between ERα and mGluR1. This is proposed to activate phospholipase C and subsequently inositol trisphosphate and the inositol trisphosphate receptor, ultimately leading to Ca^2+^-dependent N-arachidonylethanolamide (AEA) synthesis and release from the postsynapse, thereby suppressing the inhibitory effects of the synapse.

Further assessing the mechanism through which estrogen affects the synapse and therefore LTP, it was shown by light microscopic examination of immunoreactivity that the estrogen receptor α (ERα) is more abundant in the CA1 SR and the CA3 stratum lucidum of the hippocampus of female C57BL/6J mice (Mitterling et al., 2010). Moreover females have more ERα-positive synapses (Wang W. et al., 2018). This could be due to differences in membrane integration because no sex difference is detectable in cDNA abundance of ERα and estrogen receptor β (ERβ) in the hippocampus of Sprague-Dawley rats (Meitzen et al., 2019). Sex differences are also imminent regarding the function of ERα. LTP-enhancing effects of E2 in females are reduced by blocking ERα (Wang W. et al., 2018). E2 promotes an interaction between ERα and mGluR1 in inhibitory synapses of hippocampal CA1 pyramidal cells in hippocampal slices of adult female rats. This is proposed to activate phospholipase C (PLC) and subsequently inositol trisphosphate (IP3) and the inositol trisphosphate receptor (IP3R), ultimately leading to Ca^2+^-dependent N-arachidonylethanolamide (AEA) synthesis and release from the postsynapse, thereby suppressing the inhibitory effects of the synapse by inhibiting neurotransmitter release (Figure 2; Huang and Woolley, 2012; Tabatadze et al., 2015). In CA1 pyramidal cells of male Sprague-Dawley rats ERα activation replicates the frequency-enhancing effect E2 has on mEPSCs. Therefore, ERα could be involved in presynaptic mechanisms of glutamate release in males (Oberlander and Woolley, 2017). Differences in ERα signaling in males and females could be due to a different expression of proteins mediating membrane integration and interaction (Meitzen et al., 2019).

Moreover, ERβ is differentially expressed between the sexes in different brain regions. Higher levels of ERβ have been shown in males in the bed nucleus of the stria terminalis, while in females increased expression is imminent in the anteroventral periventricular nucleus of the hypothalamus near the third ventricle (Zuloaga et al., 2014). ERβ is also expressed in the hippocampus and interacts with estrogen (Smejkalova and Woolley, 2010; Kumar et al., 2015; reviewed in Hara et al., 2015). ERβ activation potentiates excitatory synapses in CA1 pyramidal cells of the hippocampus in both sexes but does so in different ways. In females the activation of ERβ sparks a presynaptic response leading to the release of glutamate (Smejkalova and Woolley, 2010; Oberlander and Woolley, 2017), while it postsynaptically modulates the actin cytoskeleton in males (Kramár et al., 2009; Oberlander and Woolley, 2017).

G-protein estrogen receptor 1 (GPER1) potentiates the synaptic response in CA1 neurons of the hippocampus in females due to an increase of postsynaptic glutamate sensitivity (Kumar et al., 2015; Oberlander and Woolley, 2017).

Testosterone can induce spine formation by interacting with androgen receptors in pyramidal neurons in the CA1 SR of male and ovariectomized female Sprague-Dawley rats. In ovariectomized females, testosterone tends to be processed to E2 by aromatase, decreasing the effect of testosterone in combination with letrozole, an aromatase inhibitor. Effects of 5α dihydrotestosterone (DHT) are visible in males and ovariectomized females independent of aromatase (Kovacs et al., 2003; Leranth et al., 2003, 2004). Androgen receptors were shown to have a lower expression in Blue-Spruce Long Evans and Sprague-Dawley rat females than in males (Xiao and Jordan, 2002; Feng et al., 2010). This effect is also visible in humans, where women show a weaker staining of androgen receptors in the lateral mamillary nucleus (LMN) and the medial mamillary nucleus (MMN) of the hypothalamus than males, even though the differences vary depending on the specific area (Fernández-Guasti et al., 2000).

### Neurotransmitters and Receptors

#### Glutamate Signaling Pathway

Glutamate is the main neurotransmitter of excitatory synapses in the mammalian central nervous system. It can activate different kinds of glutamate receptors and is involved in multiple pathways (Meldrum, 2000; Platt, 2007; Zhou and Danbolt, 2014). The activation and following integration or disintegration of glutamate receptors are important aspects in LTP and long term depression (LTD) (Lüscher and Malenka, 2012; Diering and Huganir, 2018). Because of this prominent role in the nervous system, the investigation of a potential dimorphism concerning glutamate and glutamate receptors is of significant interest.

Differences in glutamate concentrations have been investigated in multiple brain regions using magnetic resonance spectroscopy (Grachev and Apkarian, 2000; Sailasuta et al., 2008; Zahr et al., 2013). One analysis reported a slight difference between the glutamate levels of woman and man in the parietal gray matter (PGM) (Sailasuta et al., 2008). Another showed the enrichment of glutamate in the female sensorimotor cortex (Grachev and Apkarian, 2000). Average metabolite concentrations displayed an overall higher glutamate concentration in the male brain (O’Gorman et al., 2011). A different study did not find any sex-specific differences in the striatum, cerebellum and pons (Zahr et al., 2013). In the rat brain, differences in glutamate concentration exist regarding sex as well as the estrus cycle. Male rats have higher glutamate levels than females in diestrus in most brain regions among them the ventromedial hypothalamic area (VMH), the lateral hypothalamus, habenula and diagonal band. In contrast to that, female rats have more glutamate in the MPOA in all phases of the estrus cycle (Frankfurt et al., 1984). Apart from the transmitter concentration also receptors were investigated. mGluR5 was shown to have a higher expression in the PFC and the hippocampus in female, than in male rats (Wang et al., 2015).

Investigating glutamate receptors, it was shown that, some subunits of the AMPA receptor (AMPAR) differ between the sexes, while others do not. In the PSD of the hippocampus the amount of GluA1 is not affected by sex, while GluA2 and its phosphorylation at Ser880 are higher in females (Monfort et al., 2015). Also the mRNA levels of Gria2, which encodes for the GluA2 subunit, are higher in somatostatin interneurons in the medial PFC in females (Gerhard et al., 2020). AMPARs are stabilized in the postsynaptic membrane by Transmembrane AMPA-Receptor-associated Proteins (TARPs), which bind to PSD95 (Tomita et al., 2003). TARPs are therefore important for LTP, which involves an increase of AMPAR recruitment at the membrane (Tomita et al., 2005; Opazo and Choquet, 2011). In females a correlation between the binding of TARPs and the binding of SynGAP to the PSD has been shown. In the case of a heterozygous SynGAP knockout the TARP concentration increases (Mastro et al., 2020). Looking at the function of TARPs, this correlation could also influence the localization of AMPARs at the membrane.

Looking specifically at the NMDAR as the major glutamate receptor, different changes were observed between females and males. Comparing gonadectomized animals, male Sprague-Dawley rats have a higher ratio of NMDAR binding in the dentate gyrus (DG) and the CA1 stratum oriens (SO) and SR of the hippocampus than female rats. While this difference is diminished in the SR by estradiol benzoate treatment, which increases E2 levels in females, it still is imminent in the DG (Romeo et al., 2005). Looking at non gonadectomized animals over all phases of the estrus cycle higher levels of NMDA receptor (NMDAR) subunit (GluN1 and GluN2A/B) expression have been found in female Thy1-GFP mice compared to males. These differences are also imminent in cultures created from male and female Wistar rats after birth, but not in cultures generated before birth (E18). This indicates a sex specific maturation of the neurons during this time span (Brandt et al., 2020). Moreover GluN subunits of the NMDAR were shown to be increased in females compared to male Sprague-Dawley rats: GluN1 in the PFC, hippocampus and amygdala and GluN2B in the PFC (Wang et al., 2015). The knockdown of GluN2B resulted in an increase in spontaneous EPSCs in layer V pyramidal neurons of male C57BL/6J mice but not in females (Gerhard et al., 2020). In NMDAR channels GluN2B can be exchanged with GluN2A. The turnover rate concerning this change was higher in male heterozygous dysbindin mutant mice with a C57BL/6 background, due to an increase in Y1472-GluN2B phosphorylation (Sinclair et al., 2016). Regarding the role of the NMDAR, it was shown that the influence of E2 on spine formation is depending on the functionality of NMDAR in females (Woolley and McEwen, 1994; Smith and McMahon, 2006). Furthermore, low doses (0.1 mg/kg) of the NMDAR antagonist MK-801 (dizocilpine) lead to ataxia, hyperlocomotion and swaying movements of the head from side to side in female Wistar rats. Males are similarly affected at higher doses (0.3 mg/kg). This indicates a higher sensibility of females to manipulation of the NMDAR (Hönack and Löscher, 1993). Overall, the expression, excitability, and composition of NMDARs are differently affected in the sexes.

#### GABA Signaling Pathway

GABA is the main inhibitory neurotransmitter in the brain and known to be involved in multiple neurodevelopmental disorders. Moreover, many sexually dimorphic behaviors are connected to GABA signaling. Looking at aggression behavior in flies, it was shown that in male flies, female contact is recognized by the pheromone-sensing ppk29 neurons, activating a GABAergic circuit dependent on Resistant to dieldrin receptor. This pathway inhibits male-male aggression after 24 h of contact to a female fly. The GABAergic neurons in this mechanism express fruitless, a sex determinant gene, and are sexually dimorphic in number and arborization pattern (Yuan et al., 2014). This kind of dimorphisms is not only apparent in flies. In mice [C57BL/6J, gatCre/+ and Vglut2Cre/+ (Vong et al., 2011)], parenting behavior and infanticide are controlled by sexual dimorphic GABAergic neurons in the MeA. In virgin and mother females, activation of VGAT neurons can be observed in females during grooming behavior. Stimulation of these neurons leads to grooming behavior and pup retrieval in virgin females while the inhibition suppresses parenting behavior in females. In males the activation of VGAT neurons can also be observed in grooming behavior. During infanticidal behavior the same neurons show a five times higher activation. This difference can also be seen in stimulation of VGAT neurons high intensity leads to infanticidal behavior and in low intensities to grooming. This is also the case in fathers not only in virgin males. These differences might be due to differently expressed genes in male and female GABAergic neurons (Chen et al., 2019).

Also, structural differences in the brain have been connected to GABAergic neurons. The sexually dimorphic nucleus of the preoptic area (SDN-POA) is a sexually dimorphic area in the hypothalamus of rats. It is bigger in male than in female rats. By in situ-hybridization with different glutamic acid decarboxylase (GAD) oligonucleotides in albino rats of the Sprague-Dawley strain (Taconic), it has been shown that this area contains GABAergic neurons. No difference in the level of expression of the GAD isoforms was found between the sexes. In humans a similar nucleus was identified in the hypothalamus. This nucleus does contain GABAergic neurons and therefore is probably homologue to the rat nucleus. No difference was found in neural labeling between the sexes in humans. The human samples tested were formalin-fixed autopsy samples and fresh frozen samples from Alzheimer patients. This nucleus therefore provides inhibitory input to its innervation areas in rats and humans (Gao and Moore, 1996).

Specifically, synaptic differences have been studied during development and sexual differentiation. During the development of the brain, GABA can have an excitatory function. This is because Cl^–^ can move through the GABA channel in two directions; therefore, it is dependent on the concentration gradient between the cytosol and the extracellular fluid (Cherubini et al., 1991; McCarthy et al., 2002). This gradient is dependent on different Ion transporters [K^+^-Cl^–^ cotransporter 2 (KCC2), Na^+^-K^+^-2Cl^–^ cotransporter 1 (NKCC1)] and Cl^–^ channels, which change in abundance over the course of development (Plotkin et al., 1997; Kanaka et al., 2001; McCarthy et al., 2002). The hypothalamus is the region in the brain where GABA signaling becomes inhibitory first, around P6 (Obrietan and van den Pol, 1995). In other regions it can take up to P13 (Leinekugel et al., 1995; Owens et al., 1996). In the hypothalamus, excitatory GABA activity is strongest around birth. It is suspected that in the time of development GABA interacts with steroid hormones. One example supporting this theory is that, in the estrogen-receptor expressing regions (dorsomedial hypothalamic nucleus (DMH), VMH, CA1, arcuate nucleus (ARC)) of newborn mice, the GABA and GAD concentration are twice as high in male pubs compared to female ones. Other investigated regions or timepoints did not show a difference. In a different analysis it was shown that at P15 the GAD67 expression is higher in the female MeA. GAD65 showed a similar trend but was not significant. In the DMH hormonal manipulation with testosterone on P0 of castrated animals leads to higher levels of two isoforms of GAD on P1. It does not result in any differences at P15. In the MPOA independent of the testosterone treatment the GAD levels are higher in males at P1 (Davis et al., 1996, 1999; McCarthy et al., 2002). Antagonizing GABA with muscimol has opposite effects on different brain regions in neonatal male and female Sprague-Dawley rats. Phosphorylation of the cAMP response element binding protein (CREB) is increased in the MPOA, the VMH, the ARC and the CA1 area of the hippocampus in males. In females it is also increased in the ARC, but decreased in all other regions. The CA3 region shows no significant changes in both sexes upon muscimol treatment (Auger et al., 2001). In ovariectomized female Sprague-Dawley rats estradiol treatment increases GAD mRNA levels in the CA1 region and the hilus of the hippocampus. Showing the interaction of estradiol with GAD and therefore with GABA (Weiland, 1992). In the anteroventral periventricular nucleus (AVPV) of adult female Sprague-Dawley rats GABAergic neurons contain VGLUT2 mRNA. As a result of that VGLUT2 and VGAT are co-localized at synapses in the AVPV of females. These dual phenotype terminals occur rarely in male rats or in the MPOA. Moreover, an E2 treatment in combination with the afternoon decreases the number of VGAT positive terminals contacting the medial gonadotropin-releasing hormone (GnRH) neurons, which facilitate the neuronal signaling for ovulation and increase the number of dual-phenotype terminals contacting GnRH neurons. Without E2 treatment no dual-phenotype terminals contacting GnRH neurons are detectable. Overall, in E2 treated animals the immunoreactivity of VGAT goes down and the one of VGLUT2 goes up in dual-phenotype terminals in the afternoon. These experiments were done in gonadectomized males and females and show that in the female AVPV neurons can release both glutamate and GABA. These neurons are involved in luteinizing hormone surge release and ovulation, hormonal and photoperiodic signaling is necessary for that (Ottem et al., 2004).

### Neuromodulators

#### Nitric Oxide

Nitric oxide (NO) is a freely diffusible gas and serves as a cellular messenger in various organs including the brain. Glutamate, the previously discussed neurotransmitter, initiates the synthetization of NO in the central nervous system. This connection is often taken as an indicator for NOs importance in the brain (Karatinos et al., 1995; Colasanti and Suzuki, 2000; Džoljić et al., 2015). NO can have neuroprotective as well as neurotoxic effects. Therefore, it is relevant in the context of different neurological diseases (Colasanti and Suzuki, 2000; Akyol et al., 2004; Boje, 2004). All of these aspects show the importance of NO, also in the context of sexual dimorphisms.

A study specifically mapping S-nitrosylation (SNO) proteins in the cortex in female and male mice found that 266 SNO proteins were exclusively expressed in females, 320 SNO proteins in males and 189 SNO proteins were expressed in both sexes. Further analysis revealed that the SNO proteins exclusive to males were to a high extent involved in cytoskeleton remodeling and the regulation of cytoskeleton proteins in oligodendrocyte differentiation and myelination. The proteins exclusively expressed in females are involved in the regulation of Dopamine receptor 1A (D1A) signaling by glutamate and the fusion and recycling of synaptic vesicle in nerve terminals as well as activity dependent AMPAR removal at the synapse (Khaliulin et al., 2020). Apart from this overall analysis, investigations of individual proteins and pathways including nitric oxide have been done. Nitric oxide synthase-1 (NOS1) seems to be of different importance for LTP in male and female mice. In the layer IV–II/III pathway between barrel columns and in experience-dependent plasticity in the barrel cortex an αNOS1 knockout significantly reduces the probability and the magnitude of LTP in male mice, while it does not affect females (Dachtler et al., 2012). The female hippocampus contains less NO than the male one. Further on, while in males neuronal nitric oxide synthase (nNOS) levels stay unchanged, they change in females according to the stage of the estrus cycle. In proestrus and estrus the quantity of nNOS is similar to males, but it is significantly lower during diestrus and metestrus as well as in ovariectomized females. This dependency on the estrus cycle indicates a correlation between nNOS and the previously described E2. Further investigation of this correlation showed that E2 dose dependently upregulates nNOS in hippocampal neurons in females. In males the regulation of nNOS, seems to be dependent on the glucocorticoid, corticosterone system (Zhou et al., 2011; Hu et al., 2012).

#### Brain-Derived Neurotrophic Factor

Brain-derived neurotrophic factor (BDNF) is a neurotrophic factor (Barde et al., 1982) and is involved in many neuronal processes, from neuronal differentiation over plasticity and regeneration to neuronal survival (Johnson et al., 1986; Kalcheim et al., 1987; Leibrock et al., 1989; Alderson et al., 1990; Hyman et al., 1991; Ernfors et al., 1994). It is known to be differently expressed and involved in sexual dimorphic behaviors. So, the BDNF abundance in the hippocampus, the frontal cortex and the brain stem is higher in male mice over all regions (Szapacs et al., 2004; Sardar et al., 2021). Looking more closely at the hippocampus it was shown that the expression of BDNF in female Sprague-Dawley rats is higher in the CA3 region, lower in the DG and unchanged in the CA1 region compared to males (Franklin and Perrot-Sinal, 2006). This was confirmed in the right DG in male Wistar rats, showing they have more BDNF positive cells at P14 than females. No difference was found in other regions of the right hippocampus (CA1, CA2, and CA3) or in the left hippocampus. No sex differences in BDNF expression are detectable at earlier points in development (P0 and P7) (Sardar et al., 2021). In humans BDNF was found to be expressed in higher levels in the female frontopolar PFC than in the male one. Moreover, in females, the BDNF levels are lower in suicidal, depressed individuals. This data was accumulated in a western blot analysis using rapidly frozen frontopolar PFC samples of people who died through suicide or other causes (acute cardiac failure, acute myocardial infection or accident) (Hayley et al., 2015). Related to that, knocking out BDNF in the forebrain has sexually dimorphic results in mice, leading to depressive and anxiety behavior in females, while it causes hyperactivity in males (Monteggia et al., 2007). Moreover, the fact that the gene encoding for BDNF contains a section similar to an estrogen response element, could explain why BDNF is differentially regulated in males and females. The estrogen receptor-ligand complexes can bind to this sequence. Estrogen exposure increases the BDNF mRNA in the cerebral cortex and the olfactory bulb of ovariectomized rats (Sohrabji et al., 1995).

Further on, it was shown that estrogen is not the only receptor which can affect BDNF expression at the synapse. BDNF changes in abundance in response to manipulation of different synaptic receptors. In the retrosplenial cortex BDNF is regulated by NMDA. So female Sprague-Dawley rats increasing the expression of BDNF mRNA at administration of dizocilpine, an NMDAR antagonist. In males that is the case as well, but they require the administration of a higher dosage than females. Therefore, females are more sensitive to the manipulation of the NMDAR (Matsuki et al., 2001). Similarly, Phencyclidine, which also acts as an NMDAR antagonist, reduces BDNF levels in several cortical (medial PFC, motor cortex, orbital cortex, retrosplenial cortex, frontal cortex, and parietal cortex) and hippocampal (CA1 and polymorphic layer of DG) regions as well as in the amygdala (central amygdala, lateral amygdala and basolateral amygdala) and the olfactory bulb in female Lister hooded rats but not in male ones (Snigdha et al., 2011). In contrast to that male Long-Evans rats are more affected by the manipulation of GABA receptors (GABARs). This was tested by injecting pregnant rats with diazepam, a GABAR agonist. The males in the offspring were expressing higher quantities of BDNF in the hypothalamus, while males did not show a difference between diazepam and vesicle treatment (Kellogg et al., 2000). Moreover the cannabinoid agonist CP 55,940 decreases BDNF expression in the CA1 region of the hippocampus in female Wistar rats, but does not do so in males (López-Gallardo et al., 2012).

#### Neuropeptides

Neuropeptides (e.g., neuropeptide Y, vasopressin, and oxytocin) are involved in many mechanisms and pathways regulating a variety of behaviors. Sex differences are known in the behaviors regulated by neuropeptides, including stress, anxiety, feeding and social sharing (Stanley et al., 1992; Bredewold and Veenema, 2018; Ma et al., 2018; Nahvi and Sabban, 2020), as well as in the abundance of the peptides in different brain regions (Rugarn et al., 1999; Dumais and Veenema, 2016). Although there is no doubt that neuropeptides contribute to the dimorphism of the brain, since neuropeptides and the corresponding receptors are not restricted to synaptic structures and involved in volume transmission (Zoli et al., 1999; Landgraf and Neumann, 2004; Nässel, 2009; Ludwig et al., 2016), there is only limited information about the contribution of neuropeptides to the synaptic dimorphism.

It is known that vasopressin can reduce short time potentiation in glutamatergic synapses of corticotropin-releasing hormone neurons in the paraventricular nucleus of the hypothalamus of female mice. This normally is implicated in the buffering of synaptic changes by the company of a native partner after stress, but can also be induces in vitro. This was investigated using Crh-IRES-Cre; Ai14 mice, which have tdTomato expressing corticotropin-releasing hormone neurons (Loewen et al., 2020).

## Mosaic Brain and Synaptic Dimorphism

The previous paragraphs have illustrated that synaptic sex differences exist. Conclusively, one could say that synapses are sexually dimorphic. The dimorphism is depending on the investigated brain region or circuit. This variation throughout the brain could indicate that there is no universal feminization or masculinization, but a mosaic maturation of different brain areas. The idea of a mosaic brain proposes that the brain consists of several mosaic tiles, which can individually be disposed on a continuum from female to male (Joel, 2011; Joel et al., 2015). First indicators supporting this theory have been obtained in an analysis of human connectomes, showing no internal consistency in connectome and gender characteristics (Joel et al., 2015), suggesting that no dimorphism can be found on such a high organizational level. Therefore, it might make sense to look for dimorphisms on the smallest organizational level. Synapses can be taken as the smallest tiles of the mosaic, being either feminized or masculinized. The placement of a brain region on a male to female scale would then be determined by the sum of the synapses. The synaptic dimorphism is not constant among all brain regions and circuits. So, the male to female continuum seems to be specifically determined for each brain region.

## Conclusion

In this review, we provide an overview of sex differences at synapses. Besides that, there is only little data available in this field; there are strong hints that females and males differ on multiple levels, from the density of spines and synapses in different brain areas, to the abundance of specific proteins. Therefore, it is complicated to transfer the results of a study done in one sex to the other without regarding the putative sex dimorphism of synapses. Moreover, this review should illustrate that it is always important at which brain region and circuit one is looking, because sex differences vary depending on the location in the brain. This variation throughout the brain could indicate that there is no universal feminization or masculinization, but a mosaic maturation of different brain areas. The idea of a mosaic brain proposes that the brain consists of several mosaic tiles, which can individually be disposed on a continuum from female to male (Joel, 2011; Joel et al., 2015). This does not neglect the importance of gender- or sex-disaggregated data. Especially in the area of synapse analysis the data is fragmented, most of the time focusing on one specific region or circuit. So, the brain mosaic assembles bit by bit showing differences in some areas and similarities in others. That way, even if focusing on two extreme poles is not the end goal, it can help to identify regions where higher discrepancies are imminent. This might note directly indicate a binary system but a higher polarization of the continuum in this area. The larger the discrepancy the higher might be the relevance for therapeutic approaches. Moreover, dimorphisms could become relevant on the synaptic level. As synapses can be seen as the smallest anatomical tiles of the brain mosaic and we propose that they can be determined as dimorphic showing differences in several brain regions. This dimorphism would then form the continuum visible on higher organizational levels. Therefore, sex-disaggregated data is of high importance especially on low organizational levels and can be a first step toward a more individualized and differentiated approach to research and medicine.

## Author Contributions

All authors listed have made a substantial, direct, and intellectual contribution to the work, and approved it for publication.

## Conflict of Interest

The authors declare that the research was conducted in the absence of any commercial or financial relationships that could be construed as a potential conflict of interest.

## Publisher’s Note

All claims expressed in this article are solely those of the authors and do not necessarily represent those of their affiliated organizations, or those of the publisher, the editors and the reviewers. Any product that may be evaluated in this article, or claim that may be made by its manufacturer, is not guaranteed or endorsed by the publisher.
